# Stage IV Prostate Cancer Presenting With a Low Prostate-Specific Antigen Level: A Case Report

**DOI:** 10.7759/cureus.80397

**Published:** 2025-03-11

**Authors:** Brandon Weissman, Joseph l Sarow, Sumi Singh, Michael F Pusatier

**Affiliations:** 1 Otolaryngology, Lake Erie College of Osteopathic Medicine, Buffalo, USA; 2 General Surgery, University at Buffalo, Buffalo, USA; 3 Internal Medicine, University at Buffalo, Buffalo, USA; 4 Family Medicine, Buffalo Medical Group, Williamsville, USA

**Keywords:** advanced metastatic prostate cancer, prostate cancer awareness, prostate cancer (pc), psa (prostate-specific antigen), stage iv prostate cancer

## Abstract

Prostate cancer is one of the most common cancers diagnosed in men and is typically monitored through prostate-specific antigen (PSA) levels. Metastatic prostate cancer usually presents with elevated PSA levels. However, a minority of these patients have low to minimal elevation. Here, we describe the case of a 70-year-old male with a history of essential hypertension and hypertriglyceridemia who presented to the emergency department after a fall, which ultimately revealed lytic spinal lesions on computed tomography. The lesions raised the concern for possible malignancy, possible osteoporotic fractures, or multiple myeloma. Additional investigation, including a biopsy, yielded no definitive diagnosis. Notably, the patient’s PSA was 3.42 ng/mL following the non-diagnostic biopsy. Over the proceeding months, the patient developed progressively worsening weight loss and confusion. He presented to the emergency department a second time for this confusion and had a witnessed seizure. A second biopsy confirmed prostate cancer. A follow-up positron emission tomography scan further confirmed stage IV prostate carcinoma. This case highlights the complexity of prostate cancer in the face of low to mildly elevated PSA. Biological variability in prostate tumors, including the possibility of subclones that produce minimal PSA, can delay diagnosis. The patient, in this case, was started on a regimen of androgen deprivation therapy combined with abiraterone and prednisone. Clinicians should maintain a high index of suspicion for prostate cancer, especially in patients with unexplained skeletal lesions, regardless of PSA level. Clinicians should promptly perform further histopathological assessment and advanced imaging to ensure timely diagnosis and treatment.

## Introduction

Prostate cancer represented 29% of all new cancer diagnoses in men in 2024 and represents the second leading cause of cancer deaths for men in America [1]. More than 80% of men will develop prostate cancer by the age of 80 years [2]. The prostate itself is composed primarily of glandular tissue and is located in the male pelvis at the base of the penis and inferior to the bladder [3]. Prostate cancer usually begins with a mutation in the peripheral basal cells [4]. Prostate cancer is classified as adenocarcinoma, which has a characteristic glandular pattern on microscopy and will most typically metastasize to bones (84%), lymph nodes (10.6%), liver (10.2%), thorax (9.1%), and brain (3.1%) [5]. Prostate cancer metastasis to the spine is common and is often evaluated with magnetic resonance imaging (MRI). These lesions often appear as plastic sclerotic lesions but also as lytic ones. Prostate cancer is often asymptomatic in the early stage. The most common concerns are difficulty with urination, increased frequency, and nocturia. All of these symptoms can also be attributed to benign prostate hyperplasia [6]. For screening, according to the U.S. Preventive Services Task Force in 2018, men who are between the ages of 55 and 69 years should make individual decisions about prostate-specific antigen (PSA) screening [7]. PSA is a serine protease enzyme that is produced by the columnar epithelial cells of the prostate ducts and acini. Both benign and malignant prostate cells produce PSA. However, metastatic prostate cells leak more PSA into surrounding tissue, leading to more significant elevations [2]. A PSA level greater than 4 ng/mL is abnormal. Another aspect is PSA velocity, in which an annual elevation greater than 0.75 ng/mL or greater than 25% suggests potential cancer [2]. At least two abnormal PSA levels or a palpable nodule on the digital rectal exam are required to justify further investigation or biopsy [2]. Almost all patients with metastatic prostate cancer have an elevated PSA; rarely, however, a patient can present with metastatic prostate cancer with a non-elevated PSA. This occurs in less than 1% of patients with metastatic prostate cancer [8]. We present the case of a 70-year-old male with metastatic prostate cancer who presented with a low PSA level.

## Case presentation

A 70-year-old male with a past medical history of essential hypertension and hypertriglyceridemia treated with Valsartan 160 mg and cholestyramine 4 mg presented to the emergency department (ED) after a fall in March of 2024. A computed tomography (CT) scan was performed that showed a thoracic vertebra 12 (T12) burst fracture, acute lumbar vertebra 1 (L1) compression fracture with a right-sided rib fracture, and multiple lytic lesions. The hospital course was complicated by periods of aphasia, staring into space, and periods of confusion. The patient was admitted to the hospital and underwent an extensive workup with two negative MRI scans, negative electroencephalograms (EEGs), and negative CT scans. Hematology and oncology service was consulted for the lytic lesions, and a myeloma workup with serum protein electrophoresis was negative. The spine lesions were suspected to be malignant versus osteoporotic process. A biopsy was taken of the L1 lesion, which showed predominantly necrotic bone with focal fibroids and remodeling mixed with blood. An outpatient hematology-oncology appointment was recommended to the patient. After the admission, he went to subacute rehabilitation.

Once discharged from rehabilitation, the patient’s PSA was 3.42 ng/mL (reference range = 0-5 ng/mL), reassuring the medical team to determine whether the lesions were metastatic or osteoporotic. The positron emission tomography (PET)-CT was waiting to be done until the patient was more mobile and less weak. Meanwhile, the patient complained of little hunger, which was attributed to his medications.

**Figure 1:**
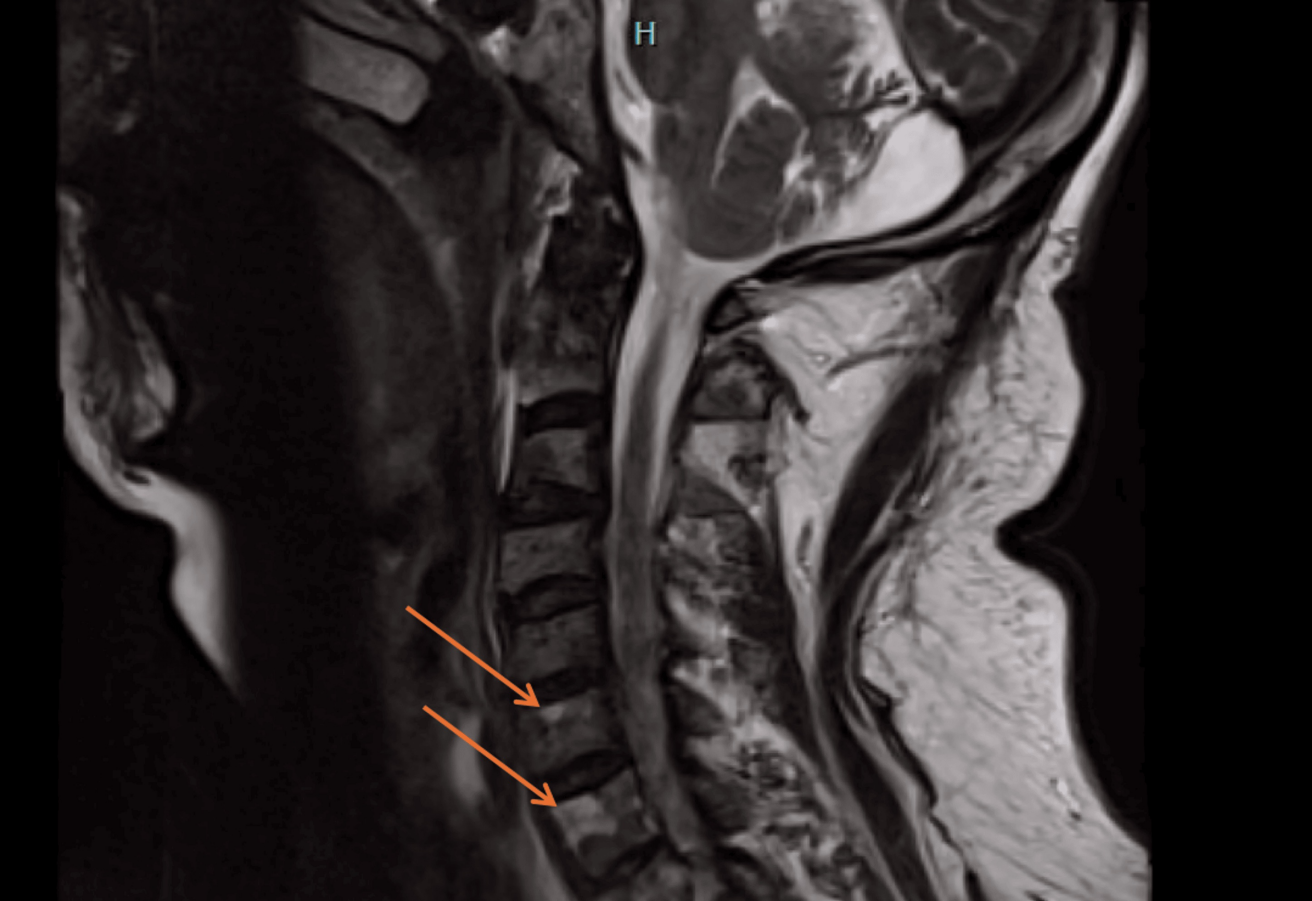
T1 sagittal magnetic resonance image of the cervical and thoracic spine. Multiple enhancing hypointense lesions throughout the cervical spine (orange arrows).

In September 2024, the patient presented to the ED with confusion, fogginess, nausea, and vomiting for the past two days. While in the ED, he had a witnessed tonic-clonic seizure that was self-limiting, and the patient was briefly postictal. Once the patient was awake, he had no recollection of the episode and no aura following the event. He was then loaded with Keppra® (levetiracetam). The patient denied any illicit drug or alcohol use, fever, dysuria, cough, or abdominal pain. The patient endorsed a 50-pound weight loss since the last admission, as well as severe pain in the lower extremities with proximal muscle weakness. The patient endorsed taking a Medrol dose pack, baclofen 40 mg, buspirone 5 mg, fluoxetine 10 mg, trazodone 100 mg, and tramadol 50 mg. The patient was subsequently admitted and underwent a full seizure workup with EEG, toxicology screen, electrocardiogram, and head CT scan. A T1 MRI showed an expansive marrow signal within the clivus, occipital condyles, and upper cervical spine, suggestive of a metastatic malignant process, and multiple hypointense lesions throughout the cervical spine, as seen in Figure 1. The patient’s alkaline phosphatase was elevated at 305 U/L. A more comprehensive list of laboratory values is presented in Table 1. During the hospital admission, the patient underwent a vertebral bone biopsy of the lytic lesion.

**Table 1:** Laboratory values on the second hospital admission .

Parameter	Result	Reference range
White blood count	8.1 K/µL	4.8–10.8 K/µL
Red blood count	3.65 K/µL	4.7–6.10 K/µL
Hemoglobin	11.2 g/dL	14.0–18.0 g/dL
Mean corpuscular volume	88.5 fL	80.0–99.0 fL
Red cell distribution width	49.0 fL	35.1–46.3 fL
Platelet count	169 K/µL	130–400 K/µL
Sodium level	133 mmol/L	133–145 mmol/L
Potassium level	3.9 mmol/L	3.3–5.1 mmol/L
Chloride level	94 mmol/L	96–108 mmol/L
Glucose level	117 mg/dL	74–99 mg/dL
Calcium level	9.1 mg/dL	8.4–10.2 mg/dL
Alkaline phosphatase	305 U/L	40–129 U/L
Ammonia	25.9 µmol/L	16–60 µmol/L

The patient was discharged with a follow-up appointment with hematology-oncology and an outpatient PET scan. The biopsy taken during the hospital admission was determined to be metastatic carcinoma consistent with primary prostate carcinoma.

In late September 2024, the patient saw oncology shortly after his second hospital admission, in which he was diagnosed with stage IV prostate cancer. The patient was put on a regimen of androgen deprivation therapy plus abiraterone/prednisone in light of the history of seizures.

In early October 2024, the patient had a PET scan, in which the scan was highly suspicious for stage IV prostate cancer with a tracer avid mass in the apex of the prostate gland, with innumerable lytic bone metastases and two metastatic lymph nodes in the left pelvis. He remains in treatment for prostate carcinoma.

## Discussion

Prostate cancer represented nearly one-third of all cancer diagnoses in men in 2024 [1], with the majority of men receiving a prostate cancer diagnosis in their lifetime. It is vital to have a robust screening protocol. Most patients with metastatic prostate cancer will have an elevated PSA; however, there is a rare subset of these patients who present with little to no elevation in PSA [9]. In this case report, we presented a patient whose PSA was 3.42 ng/mL after presenting with lytic spinal lesions during his first hospital admission. This is below the PSA level of 4 ng/mL, which raises clinical suspicion. Despite this patient’s relatively low PSA, they were ultimately diagnosed with stage IV prostate cancer, which emphasizes the importance of including prostate cancer in the differential even with PSA levels below the typical level of suspicion.

Prostate cancer is biologically diverse and often multifocal [8]. Higher-grade tumors are more prone to developing a clonal shift, and, by doing so, they could adopt characteristics of other prostate cancers, such as greater aggressiveness that fails to produce PSA. This phenomenon of tumor progression without a corresponding rise in PSA can be explained by the growth of cell lines that do not generate PSA or poorly differentiated cells that have lost the ability to produce PSA [8].

In the presented case, imaging and histopathological confirmation proved critical to the final diagnosis. The patient’s fall and broken ribs led to the discovery of lytic lesions, which prostate cancer causes, but also many non-cancerous conditions. The biopsy results showed necrotic bone, which led our medical team without a definitive diagnosis. After discharge from the hospital, the patient went to outpatient rehabilitation and was not ready to get the PET scan, as the patient felt too weak. Unfortunately, while awaiting the PET scan, the patient presented for his second admission with confusion and a witnessed seizure. At this time, a second biopsy was attained, which confirmed the diagnosis of prostate carcinoma, and a PET scan affirmed the diagnosis of stage IV prostate cancer.

Therapeutically, the patient was prescribed a regimen of androgen deprivation therapy plus abiraterone/prednisone, which is consistent with current guidelines for advanced prostate cancer. Androgen deprivation therapy works in the treatment of prostate cancer due to prostate cancer cells’ reliance on oncogenic androgen receptor signaling. The treatment profoundly reduces testosterone and estrogen levels, leading to a significant anticancer response [10]. The patient’s seizure history influenced treatment choice as some hormonal and supportive medications can induce neurological effects.

In summary, this case underscores the need for clinicians to have a high index of suspicion of prostate cancer with unexplained skeletal lesions, even in the face of a standard or mildly elevated PSA level. Early biopsy and comprehensive imaging, including PET scans, are crucial for accurate diagnosis and timely initiation of appropriate therapy.

## Conclusions

This case provides critical insights into the need for a high index of suspicion for prostate cancer even when PSA levels are not markedly elevated. While PSA screening remains a vital tool, due to the biologically diverse nature of prostate cancer, certain subtypes can produce minimal PSA, resulting in little to no elevation. Early consideration of prostate cancer, coupled with prompt histopathological assessment and advanced imaging, is crucial for accurate diagnosis and timely initiation of therapy in patients with unexplained lytic bone lesions and potential metastatic disease.
